# A Mobile Multimedia Reminiscence Therapy Application to Reduce Behavioral and Psychological Symptoms in Persons with Alzheimer's

**DOI:** 10.1155/2018/1536316

**Published:** 2018-03-21

**Authors:** Danish Imtiaz, Arshia Khan, Adriana Seelye

**Affiliations:** ^1^George Washington Medical School, Washington, DC, USA; ^2^Computer Science Department, University of Minnesota Duluth, Duluth, MN, USA; ^3^University of Minnesota Twin Cities, Minneapolis, MN, USA; ^4^Minneapolis Veterans Affairs Healthcare System, Minneapolis, MN, USA

## Abstract

The goal of this project is to develop a novel and innovative mobile solution to address behavioral and psychological symptoms of dementia (BPSD) that occur in individuals with Alzheimer's. BPSD can include agitation, restlessness, aggression, apathy, obsessive-compulsive and repetitive behaviors, hallucinations, delusions, paranoia, and wandering. Alzheimer's currently affects 5.4 million adults in the United States and that number is projected to increase to 14 million by 2050. Almost 90% of all affected with AD experience BPSD, resulting in increased healthcare costs, heavier burden on caregivers, poor patient outcomes, early nursing home placement, long-term hospitalizations, and misuse of medications. Pharmacological support may have undesirable side effects such as sedation. Nonpharmacological interventions are alternative solutions that have shown to be effective without undesirable side effects. Music therapy has been found to lower BPSD symptoms significantly. Our study is based on combination of the reminiscence and the music therapies where past memorable events are recalled using prompts such as photos, videos, and music. We are proposing a mobile multimedia solution, a technical version of the combined reminiscence, and music therapies to prevent the occurrence of BPSD, especially for the rural population who have reduced access to dementia care services.

## 1. Introduction

Approximately 35.6 million people worldwide and more than 5 million in the United States suffer from dementia. Every 66 seconds, a person in the United States develops dementia [1]. Almost 90% of all affected with Alzheimer's disease (AD) experience behavioral and psychological symptoms of dementia (BPSD), resulting in increased healthcare costs, heavier burden on caregivers, poor patient outcomes, early nursing home placement, long-term hospitalizations, and misuse of medications [2]. BPSD play a major role in increasing burden on the caregivers of individuals affected with dementia [3]. BPSD is not only a contributor to caregiver distress but also a major cause for nursing home placement [4]. As the number of affected people increases, the need for caregiver also increases. More than 15 million family and friends of affected people provide care for the person affected with AD. In 2016, over $236 billion dollars were spent on dementia [1]. Indirect cost increase due to BPSD in person affected with AD was 25% above the cost of AD, while the direct costs increase was 35% above the direct AD costs [4].

As the disease progresses, many affected individuals develop psychological problems in the form of behavior issues such as agitation, irritability, aggression, depression, delusions and hallucinations, wandering, and sleep disorders [5–7]. Among behavioral symptoms, approximately 88% of the patients suffer from apathy, 66% suffer from aggression, and 60% suffer from irritability, while among psychological symptoms, 56% of patients suffer from depression, 55% suffer from delusions, and 52% suffer from anxiety. In one study, not only were behavioral symptoms found to be more prevalent, but these symptoms were also more stressful to caregivers than the psychological symptoms [7]. If these symptoms are not treated, they can reduce the quality of life, expedite functional decline, and cause early transfer to assisted living [1, 7, 8]. Of these symptoms, the most prevalent and distressing were behavioral symptoms such as agitation, irritability, and apathy [9]. The stress process model identifies BPSD as the greatest source of stress on caregivers [10]. Persons with mild to moderate AD who are affected with BPSD often require frequent supervision, monitoring, and support. This poses several challenges, especially in rural parts of the country due to limitations in resources. Approximately 30% of the total costs for AD are from BPSD management [4]. As per the US census of 2010, approximately 19.3% of the US population resides in rural areas that covers 97% of the land [11]. Due to the large distances between the rural communities, the residents in rural areas are faced with greater health care barriers than their urban counterparts. Some of the barriers experienced by the residents in rural areas are constrained access to care, limited resource availability, overlapping clinician roles, long distance provider commute, and clinician training constraints [12]. Rural residents face problems of not only scarcity of primary care providers but also long distances between care providers and the rural communities [13]. In addition, rural areas have a proportionally larger elderly population (14.6% of the rural population is 65 and above versus 11.7% of urban population) and hence a larger number of individuals affected with dementia and fewer health and social services. The goal of this project is to create a calming and relaxing effect while helping resurface embedded event memory in persons with AD by tackling distressing BPSD symptoms. Pharmacological support in the form of antipsychotic drugs can be used, but these are not always helpful and may have side effects such as sedation. Nonpharmacological solutions such as reminiscence therapy and music therapy are encouraged in addressing BPSD.

Music therapy has proved to lower BPSD in persons affected with dementia. In one study, music therapy was applied for 16 weeks and subjects were evaluated by multidimensional assessment including minimental state examination, Barthel index, and neuropsychiatry inventory. These subjects were observed to have a significant decrease in the NPI score, and the symptoms of agitation, irritation, apathy, delusions, and hallucinations were greatly reduced [14]. Reminiscence therapy has been successfully used to help address BPSD [15]. However, traditional reminiscence therapy is time consuming, which makes it difficult to implement, especially in a rural setting. Additionally, this type of therapy is conducted in a group setting and requires resources such as a trained group leader and other members who are skilled to work in this group. These resources are often not available to community-dwelling older adults who live in rural areas.

**Figure 1:**
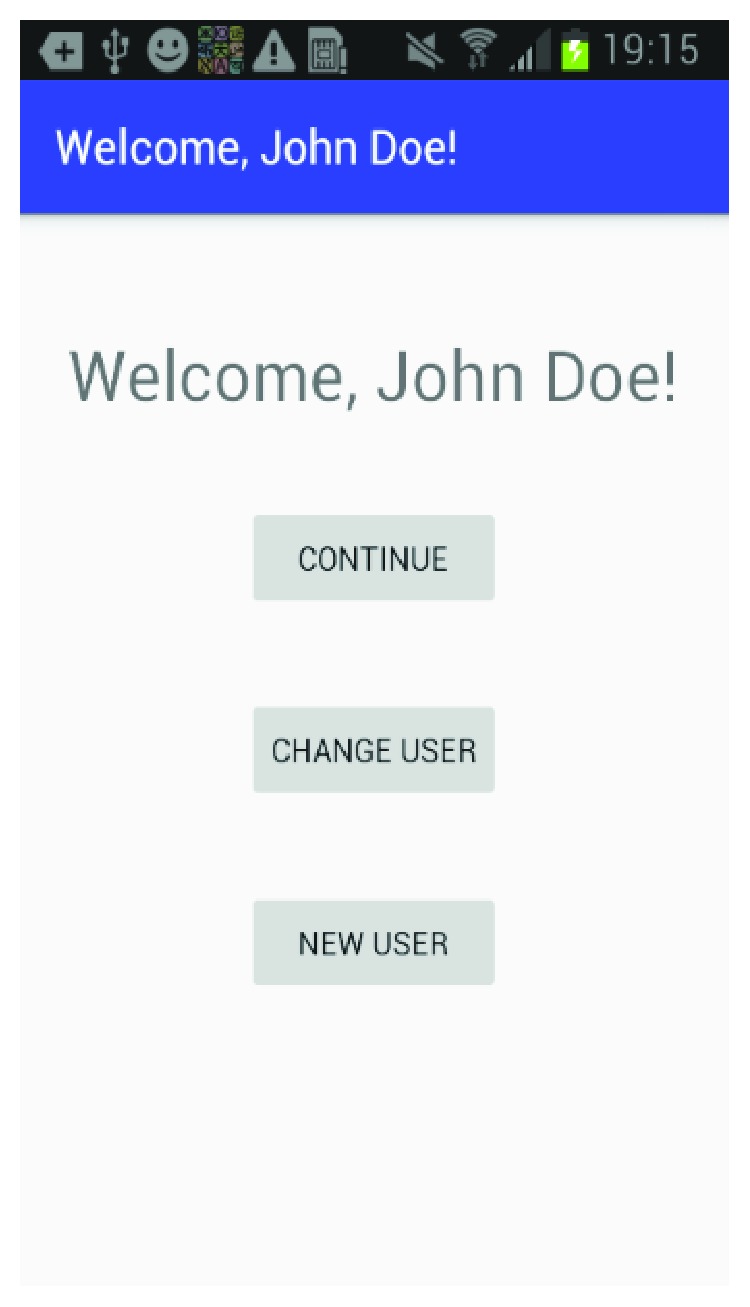
Welcome page.

**Figure 2:**
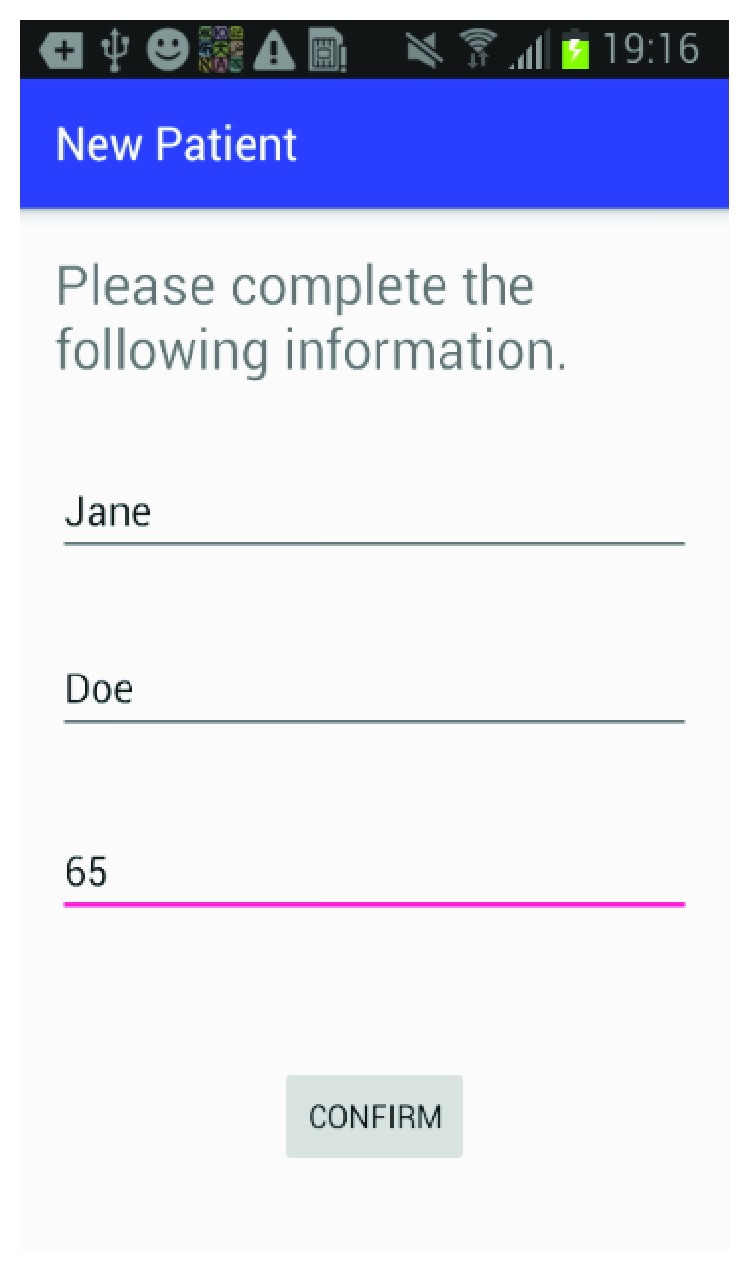
Patient information.

**Figure 3:**
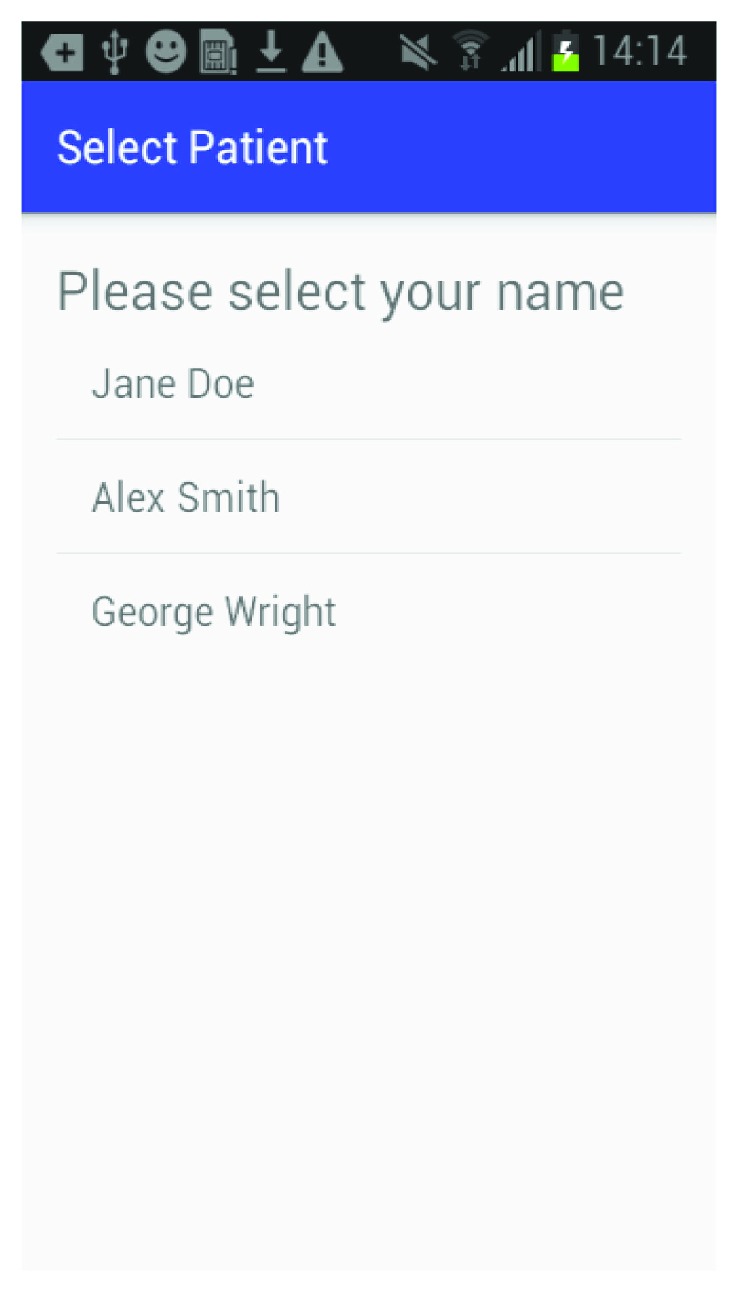
Select patient.

**Figure 4:**
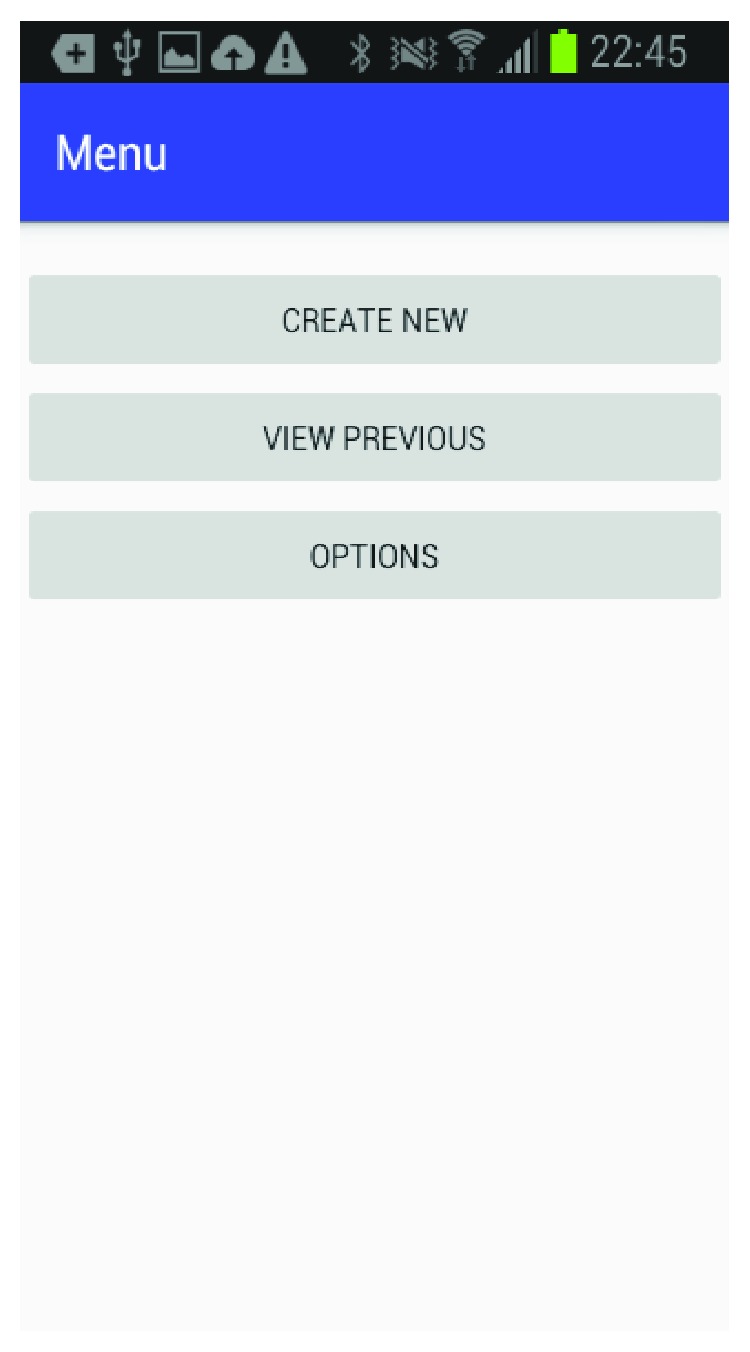
Menu selection.

**Figure 5:**
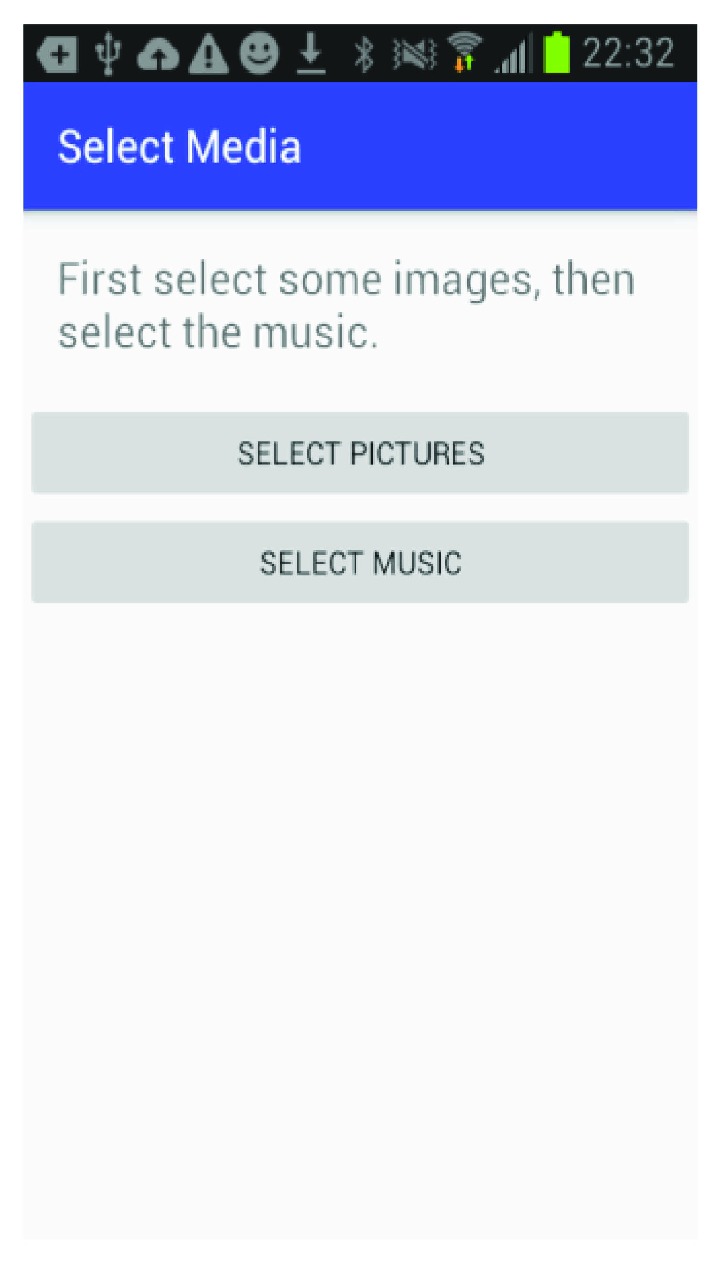
Media selection.

**Figure 6:**
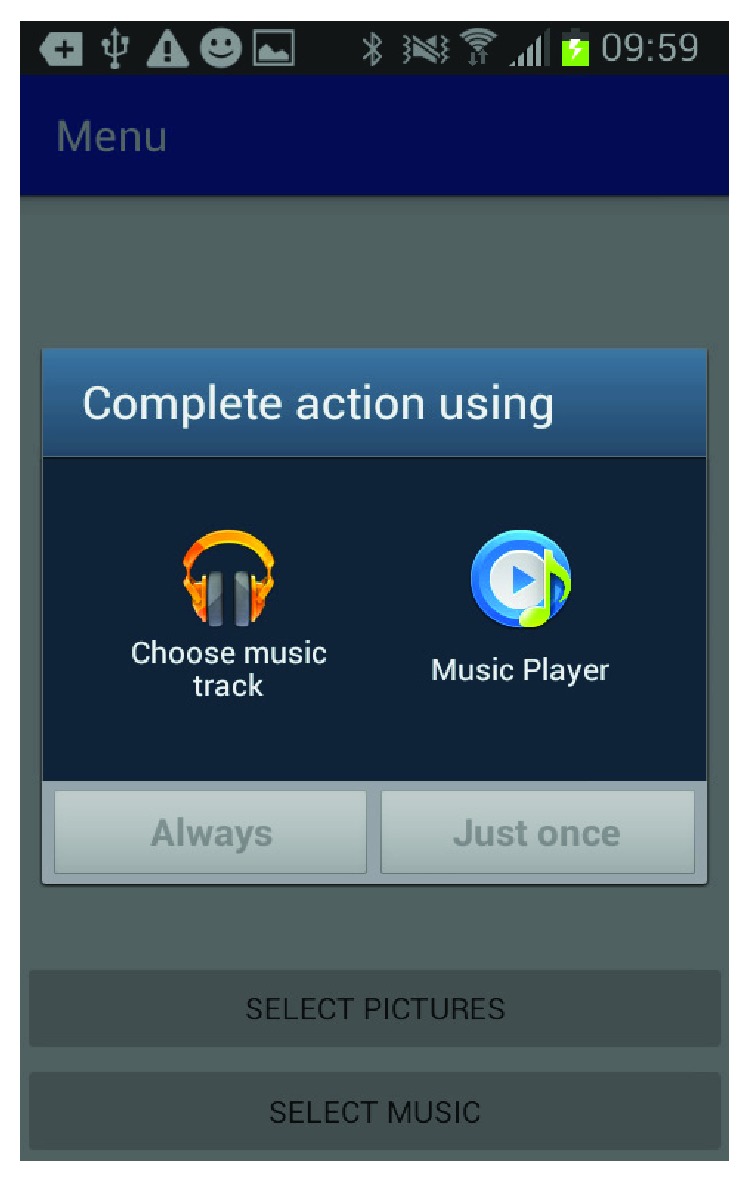
Music source.

**Figure 7:**
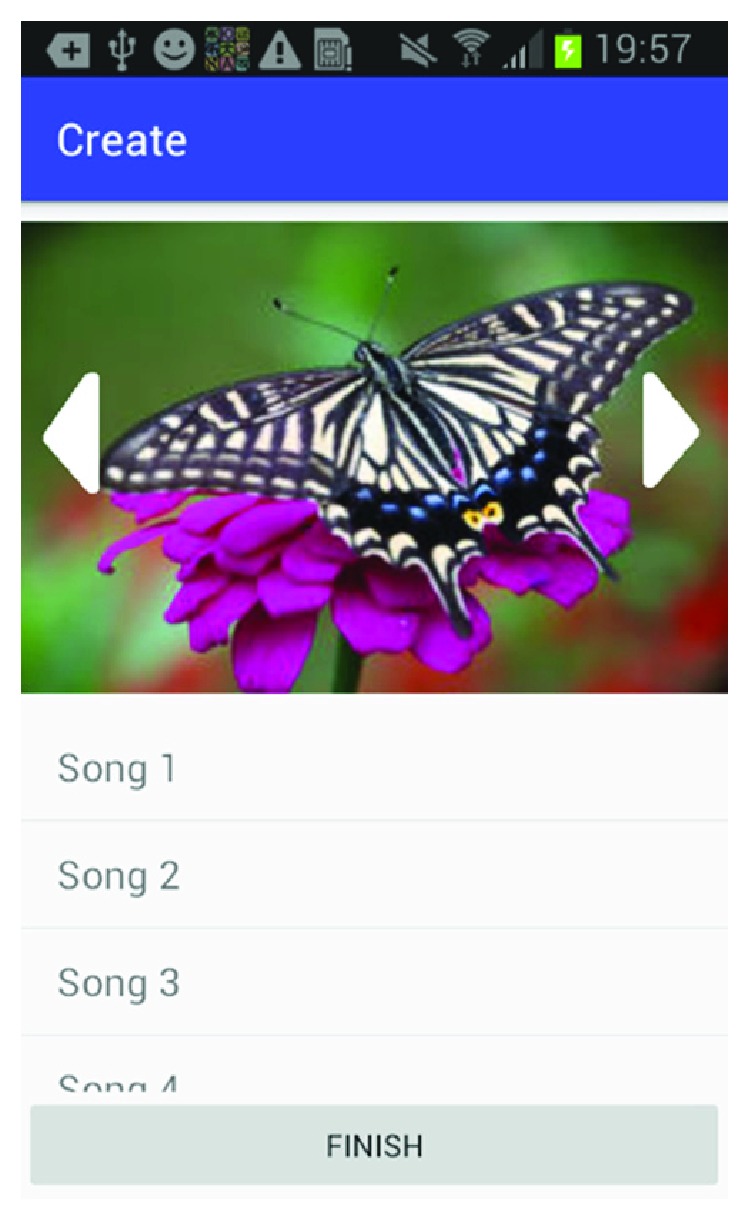
Music selection.

**Figure 8:**
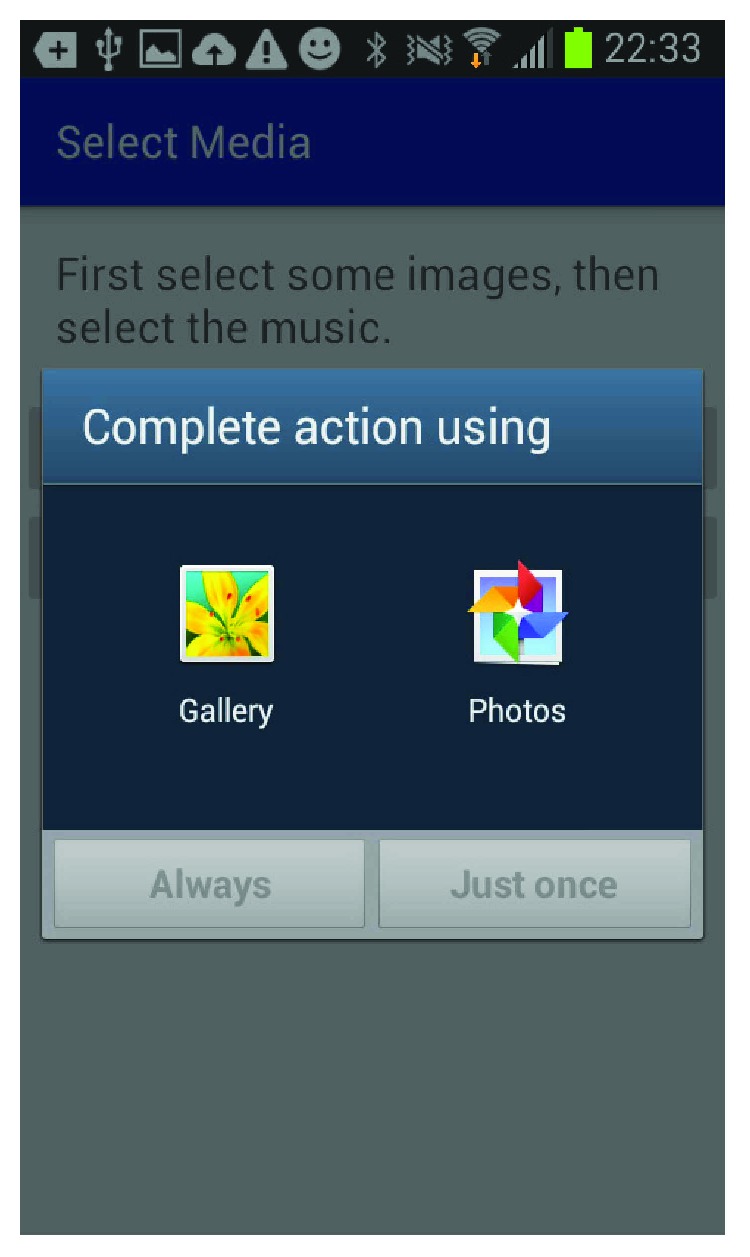
Photo source.

**Figure 9:**
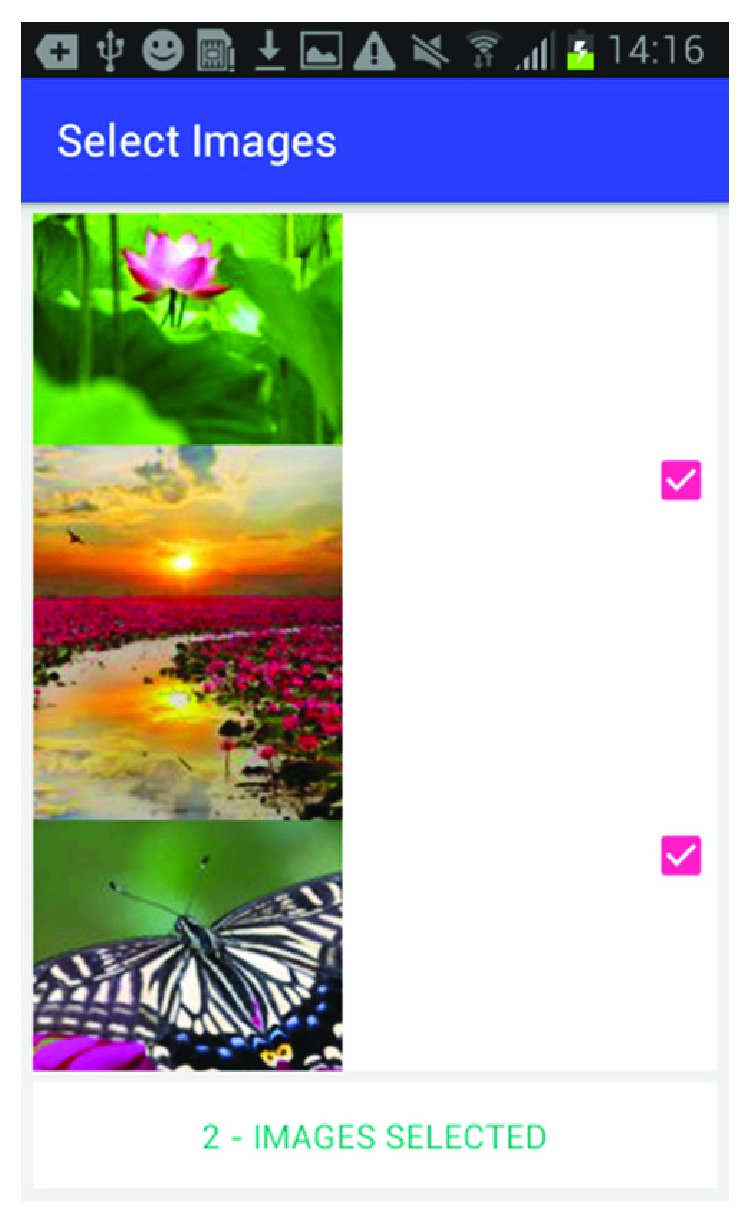
Photo selection.

**Figure 10:**
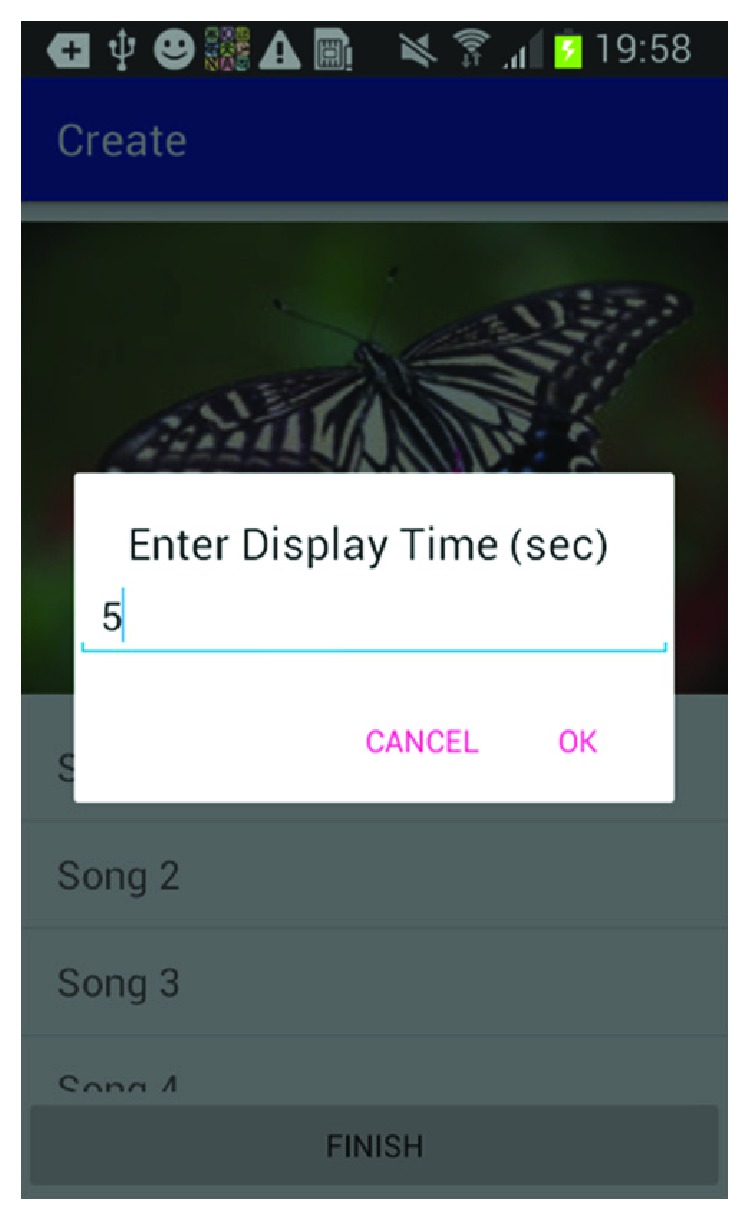
Photo display time.

Our proposal is based on two foundational concepts—reminiscence therapy and Dr. Oliver Sack's music research. Reminiscence therapy is used to recall past memorable events using prompts such as photos and music in group settings where a group leader would lead the session with other friends and family of the affected individual and the event is gently reminded with cues from the event. Although traditional reminiscence therapy has proved to help lower BPSD symptoms notably, it is not only time and resource intensive; it is typically conducted in a group setting and requires resources such as a trained group leader. We are proposing a variation of the reminiscence therapy where the group leader and the other family members are not required to be present each time the therapy is performed. Dr. Oliver Sack, a neurologist, has promoted music as a powerful tool to stimulate and recall deeply embedded memory [16–20]. Irrespective of their stage in AD, all people are able to respond and react to musical stimulation in improving cognition, behavior, and mood in AD [18]. Research has shown individualized music therapy is the most effective [19]. Our proposal creates an individualized multisensory mobile multimedia environment that is tied to a specific memory event as seen in Figures 1, 2, 3, 4, 5, 6, 7, 8, 9, and 10.

## 2. Related Work

Technology has been proposed as a means of treating many facets of dementia, including challenging behaviors such as anxiety, restlessness, agitation, sleep disturbances, and disorientation, especially in regard to time and place and physical functioning [21, 22]. As can be seen, the scope of these technological innovations varies greatly. In addition to the scope, the approach utilized by these applications differs significantly. Among the investigated applications are care-team communication tools, electronic encyclopedias, decision support applications for at-home caregivers, Internet-based support groups, home monitoring systems, human-to-human interaction tools, computer-based memory tools, audio and video tapes, and entertainment [21–24]. Of all the related technological innovations, two stood out as strongly related to the proposed application. The first, developed by a research team made up of professionals from the National Research and Development Centre for Welfare and Health from Finland, Dementia Services Information and Development Centre from Dublin, the Norwegian Centre for Dementia Research, and Dementia Voice from the United Kingdom evaluated the effectiveness of a music-based and multimedia program in dementia care centers in the four represented countries with the primary aim of stimulating patients and to give them pleasure. This was a word processing editor called the picture gramophone and designed for a desktop with a CD drive, a keyboard, and a mouse. Results showed that 91% of participants showed some form of benefit from the tool, in either mood or social interaction [25]. The application design varied from our proposed solution in that it was specialized to the region and minimally modified to the individual but in no way individualized to the particular patient or events in that patient's life, although the authors noted that this should be a consideration for future developments. In addition, because of logistics, this technology was available to patients only at the care center. Finally, some patients needed support when working with the tool. The second application, Snoezelen, is a multisensory environment that seeks to reduce agitation and anxiety or stimulate reactions of communication in patients by creating a very engaging yet controlled environment. Snoezelen has been used to reduce challenging behavior among individuals affected with AD [26, 27]. Various studies have shown improvement in mood, behavior, and cognition after treatments. Patients' communication improved during the Snoezelen sessions, and patients showed improvements in both short- and long-term follow-up evaluations [28]. These studies illustrate the impact technology can have on management of BPSD and improving the lives of those with dementia and their caregivers. The picture gramophone, a word processing solution, was a music-based multimedia tool that was developed in the 1990s to help improve the well-being in persons with dementia and displayed promising results [25].

Current technological solutions and support applications for caregivers do not address BPSD directly. The objective of our multimedia application is to stimulate deeply embedded memories by recreating events through use of music, images, and textual descriptions, for example, incorporating pictures of important events such as a wedding or birthday into a slideshow to reduce BPSD in persons with Alzheimer's disease. The objective of our project is to address BPSD in persons with AD by stimulating deeply embedded memories by recreating events through use of meaningful music and personalized images and textual descriptions. For example, we will incorporate pictures of important events such as a wedding or birthday into a slideshow that can calm the affected individual.

## 3. Framework

We are proposing a framework to address BPSD in dementia such as agitation, irritability, and apathy by combining therapies such as reminiscence and music therapies into a technological, affordable, personalized, and accessible mobile solution to reduce BPSD and their associated stress on caregivers. The uniqueness of this proposal is the design with respect to accessibility and affordability of a mobile application and the individualized nature of the music and reminiscence therapy components. The caregiver can pick an important event in the life of the affected individual and use cues, such as pictures, videos, and music, associated with that event. Cues help jog deeply embedded memories related to an event; for example, incorporating pictures of important events such as a wedding or birthday into a slideshow can help the affected individual to calm down. Reminiscence therapy and music therapies have proved to be very beneficial in addressing BPSD and have been recommended to reduce BPSD. We are combining the concept of using prompts such as objects and people in helping recall episodic-embedded memory from reminiscence therapy and the concept of music helping in memory recall in persons affected with AD as promoted by neurologist, Dr. Oliver Sack. We are proposing to incorporate the positive aspects of reminiscence and music therapies into a user-friendly mobile application that is affordable and easily accessible to caregivers and persons with AD. Pharmacological solutions are not encouraged due to the undesirable side effects of sedation. Our proposal is a low-cost solution to address BPSD and help support caregivers. The mobile app will be designed for multiple mobile platforms such as iOS and Android. The challenges in this application will be the ability to select music/video/pictures from the mobile user's music playlist/photo/video library, or if there is no music associated with this particular event, and then performing a search to find music of the era of the same timeframe as the event for which the multisensory multimedia event is being created. Another challenge will be to design the user interface in a way that is user-friendly and accessible to the caregiver, who will most likely be an older adult and who may or may not have much computer or smartphone experience. Also, it will be important to make the music/picture selection process a quick one, as caregivers will likely want to address BPSD symptoms quickly once they occur. The caregiver will decide on an event such as a wedding and find pictures, videos, and music-associated with this event. These will be incorporated into a multimedia slideshow and played to the affected individual when he/she is agitated, irritated or experiences apathy.

## 4. Methods and Design

In this two-phase exploratory study, a multisensory multimedia-based mobile application has been developed for the Android mobile platform as seen in Figures 1, 2, 3, 4, 5, 6, 7, 8, 9, and 10 for phase 1, while the clinical study will be conducted in phase 2. Only phase 1 will be described in this paper. Phase 1 will comprise of designing and building the mobile application for multiple platforms, and phase 2 will comprise of the clinical study. The objective of phase 2 of the project is to stimulate deeply embedded event-based episodic memories of persons affected with AD. The caregiver will pick an important memorable event from the life of the affected individual and use cues, such as pictures and music, associated with that event to create a multisensory multimedia show paired with music that was either from the event or associated with the event. If there is no music associated with this event, then the application will find music of that period to create this multimedia show. The videos or photos will serve as prompts and cues to help jog deeply embedded memories related to this event. An example of creating a multisensory multimedia show is selecting pictures and videos of important events such as a wedding or birthday into a slideshow that can help the affected individual to calm down. This calming effect potentially helps the affected individual recall associated memories. The project will be executed in two phases. The design is built on the following research question and hypotheses.

Research question: Can a multisensory mobile multimedia reminiscence therapy app reduce the frequency of BPSD episodes by helping calm and reducing agitation, irritation, and apathy by recreating an event/episodic memory in persons with AD?

### 4.1. Phase 1/Aim 1

Design a multisensory mobile application that employs reminiscence therapy to reduce the frequency of BPSD episodes in persons with AD.

Hypothesis: After engaging with the reminiscence therapy app during an episode of BPSD, there will be a reduction in neuropsychiatric symptoms as evidenced by caregiver-report ratings on a semistructured questionnaire. There will be an overall reduction in neuropsychiatric symptoms over the one-week period as evidenced by lower scores on validated caregiver-report questionnaires assessing BPSD such as the neuropsychiatric inventory (NPI; Cummings, 1994) and the revised memory and behavior checklist (RMBC; Terri, 1992).

### 4.2. Phase 2/Aim 2

Design a multisensory mobile application that engages the person with AD and reduces caregiver burden.

Hypothesis: After engaging with the reminiscence therapy app during an episode of BPSD, there will be a reduction in caregiver distress as evidenced by caregiver self-report ratings on a semistructured questionnaire. There will be an overall reduction in caregiver distress over the one-week period as evidenced by lower scores on validated self-report questionnaires assessing BPSD-related caregiver distress such as the NPI and the RMBC.

The mobile application is built for the Android platform and is designed to be user-friendly so a caregiver can easily set up a patient and create the multimedia episodic happy memory event-based presentations for the patients.  Figure 1 shows the welcome page of the application when it firsts starts; Figure 2 gives user the option of entering the information for a new patient as this application can be used for multiple individuals affected with AD; Figure 3 shows the screen where an existing patient can be selected from a database of patients; Figure 4 shows where the caregiver has the choice of either choosing to create a new happy memory multimedia presentation or selecting from a list of happy memories that were created in the past; the happy memory events created are unique to each patient with their photos and music. Hence, this application can be customized to each individual's needs making unique set of episodic happy memories.  Figure 5 shows the first screen that appears when the caregiver chooses to create a new happy memory presentation, where the caregiver has a choice of either selecting pictures for the new happy memory or music for this new presentation since various pictures and or videos can be incorporated in the multisensory multimedia event-based presentation.

Figure 6 shows how the caregiver can select between music databases to add music associated with this happy memory.  Figure 7 shows the screen where the caregiver can select the music from the music library.  Figure 8 shows the screen for the photo selection while Figure 9 shows where the images/photos are selected. Finally, Figure 10 shows how the user can select the length of time of display for the images/photos.

At clinical study (phase 2) entry, caregivers will complete validated self-report measures assessing BPSD in their AD person over the past week and BPSD-related caregiver distress. Caregivers will provide study staff with pictures, movies, music, and any other media that is meaningful to the AD participant's past to be loaded on the multimedia app. After the media has been loaded on the study tablet, participants will be given the tablet equipped with the multimedia application to use in their home for one week. Any time the AD participant experiences BPSD during the one-week study period, the caregiver will attempt to engage the participant with the multimedia app and then rate the experience using a brief self-report questionnaire. At clinical study exit, caregivers will repeat the validated self-report questionnaires and complete a feedback interview. The goal of the feedback interview is to break down the user experience and solicit likes and dislikes and attitudes about the multimedia app solution.

**Figure 11:**
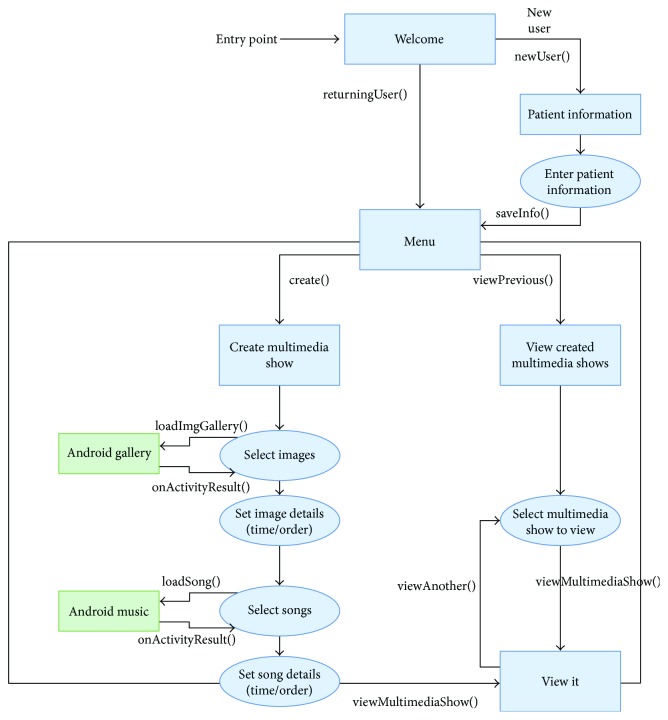
Algorithm for the *Happy Times* Android application.

The algorithm for this study is shown in Figure 11, where the application starts with a welcome screen and the user/caregiver has the option of either creating a new patient or selecting a patient that has been registered in the past. Next, the user is taken to a menu where the happy memory presentation can be created or a previously created happy memory event presentation can be viewed. The user then has a choice between selecting music or photos that can be added to the happy memory. The algorithm shows the path for this process where the Android gallery and the Android music is browsed for the multimedia selection.

## 5. Discussion

Persons with mild to moderate AD who experience BPSD often require frequent supervision and monitoring, posing several challenges to their caregivers. The stress process model identifies BPSD as the greatest source of stress on caregivers, with sleep disturbance, agitation/restlessness, depression, and apathy causing the most emotional distress. We are proposing a multisensor mobile multimedia solution, a technical version of the traditional reminiscence therapy to address BPSD, especially for the rural AD population. Rural areas have a proportionally larger elderly population (14.6% of the rural population is 65 and above versus 11.7% of urban population) and hence a larger number of individuals affected with dementia and fewer health and social services [29–32].

After completion of phase 2, descriptive statistics (means, standard deviation, and frequencies) will be used to describe the sample characteristics, total scores, and/or the item-level results of the clinical tests, validated survey instruments (NPI; RMBC), and the caregiver ratings of BPSD symptoms and caregiver distress after engaging with the multimedia app. For instruments that are administered at study entry and exit, we will compare each instrument's total score at the beginning and the end of the one-week period using dependent *t*-tests with false discovery rate correction for the number of variables. To examine feasibility of deploying the app preloaded on tablets in participants' homes for use over one-week, we will track and report the following data: (1) the percentage of participants who were unable or unwilling to multimedia app; (2) the number and type of technical or other problems with the multimedia app reported by participants; and (3) the number of phone calls and/or unanticipated home visits required by research staff to troubleshoot problems with the multimedia app. Study personnel will document any problems reported by participants. If participants are unable or unwilling to complete the in-home study after consenting, we will examine whether there are differences in demographic variables (e.g., age, education, and ethnicity) or global cognition (total MoCA score) between study completers and noncompleters using independent *t*-tests for continuous variables and the Pearson chi-square test for categorical variables.

The long-term goal of this research is to develop a mobile solution to (a) reduce the frequency of BPSD symptoms, (b) help recall episodic memory, (c) reduce caregiver burden, and (4) delay nursing home placement.

## 6. Conclusions

Our interdisciplinary team of computer scientists and a clinical neuropsychologist are working together on developing a tool to help individuals affected with AD and their caregivers cope with behavioral and psychological symptoms of dementia. The application is designed to help calm individuals with AD by jogging old episodic memories and bringing back memories of happy times.

This proposal is built by combining the foundational concepts of the reminiscence therapy along with the concept of music research conducted by Dr. Oliver Sack. Reminiscence therapy has been successfully implemented to recall past memorable episodic events using prompts and cues such as photos, in group settings where a group leader would lead the session with other friends and family of the affected individual and the event is gently reminded with cues from a past episodic event. We have created a technical version of the reminiscence therapy in the form of a mobile application that is built for the Android platform. Dr. Oliver Sack, a neurologist, has promoted music as a powerful tool to stimulate and recall deeply embedded memory [16–20]. His research has demonstrated that irrespective of the stage in AD, all affected people are able to respond and react to musical stimulation in improving cognition, behavior, and mood in AD [18]. Research has shown individualized music therapy is the most effective [19]. Our proposal creates an individualized multisensory mobile multimedia environment that is tied to a specific memory event.
